# HANDLSleep: Associations among neighborhood quality, food security, and cognitive function

**DOI:** 10.1002/alz70860_098034

**Published:** 2025-12-23

**Authors:** Alexa C Allan, Orfeu M. Buxton, Lesley A. Ross, Christopher G. Engeland, Roland J. Thorpe, May A Beydoun, Alan B Zonderman, Michele K Evans, Alyssa A. Gamaldo

**Affiliations:** ^1^ The Pennsylvania State University, University Park, PA, USA; ^2^ Clemson University, Institute for Engaged Aging, Clemson, SC, USA; ^3^ Johns Hopkins Alzheimer's Disease Resource Center for Minority Aging Research, Bloomberg School of Public Health, Baltimore, MD, USA; ^4^ Laboratory of Epidemiology & Population Sciences, Baltimore, MD, USA; ^5^ Clemson University, Institute for Engaged Aging, Seneca, SC, USA

## Abstract

**Background:**

Previous research examined the separate roles of neighborhood quality (Chen, Lee, & Huang, 2022) and food security (Na et al., 2020) on cognitive function among older adults. However, there are few investigations of the combined roles of neighborhood quality and food security on cognition. Thus, the current study examined the cross‐sectional association among neighborhood quality, food security, and cognitive function.

**Method:**

This study included 220 community‐dwelling, socioeconomically diverse Black and White adults (M_age_ = 62.74, SD_age_ = 8.84; 71% female) from the Healthy Aging in Neighborhoods of Diversity across the Life Span Sleep (HANDLSleep) study. Neighborhood quality was assessed using the Neighborhood Atlas Area Deprivation Index and self‐reported measures of physical built disorder (e.g., litter), social cohesion (e.g., close‐knit neighborhood), and social control (e.g., neighbors act if fight breaks out). Food security was assessed with the item, “Do you have enough money for the kind of food you or your family should have?”. Cognitive functioning was assessed using a neuropsychological battery including memory, learning, attention, processing speed, and executive function measures. Multivariable linear regression analyses were conducted with each cognitive measure as the outcome. Models were adjusted for age, sex, race, reading literacy, poverty status, depressive symptoms, and medical condition history (e.g., diabetes, hypertension).

**Result:**

In the adjusted models, food security was associated with better performance on a measure of visuospatial skill (*p* < .05). Significant interactions were observed between neighborhood quality and food security. Specifically, high food security was associated with faster performance on a measure of executive function and processing speed for participants living in less disadvantaged neighborhoods (state level: b = ‐16.85, SE = 6.12, *p* = 0.02). Among individuals living in less physical built disorder, high food security was associated with better global cognitive status (b = 2.28, SE = 0.93, *p* = 0.03). Among individuals living in neighborhoods with better social cohesion, high food security was associated with better performance on a measure of visuospatial skill (b = 1.85, SE = 0.50, *p* = 0.004).

**Conclusion:**

These findings underscore the importance of examining the combined effects of neighborhood quality and food security on cognitive function.

